# Orange‐fleshed sweet potato (*Ipomoea batatas*) composite bread as a significant source of dietary vitamin A

**DOI:** 10.1002/fsn3.543

**Published:** 2017-11-20

**Authors:** Victoria Awuni, Martha Wunnam Alhassan, Francis Kweku Amagloh

**Affiliations:** ^1^ Department of Food Science and Technology University for Development Studies Tamale Ghana

**Keywords:** bread, orange‐fleshed sweet potato, vitamin A, wheat flour

## Abstract

Refining food recipes with orange‐fleshed sweet potato (OFSP) has the potential to improve dietary intake of vitamin A. The objectives of this study were to utilize OFSP in the development of two composite bread types and to assess their contribution to dietary intake of vitamin A using the dietary reference intake of lactating mothers. Two composite OFSP–wheat flour bread recipes—vita butter bread and vita tea bread—were developed by incorporating 46% OFSP puree in existing 100% wheat flour bread recipes consumed by Ghanaians. A paired‐preference test was used to profile the appearance, aroma, sweetness, and overall degree of liking of the vita butter bread and vita tea bread and their respective 100% wheat flour bread types. Weighed bread intake by lactating mothers (*n *=* *50) was used to estimate the contribution to dietary vitamin A based on the trans β‐carotene content. The developed vita butter bread and vita tea bread were most preferred by at least 77% (*p *<* *.05) of consumers (*n *=* *310) for all the attributes considered. The average daily intake by the lactating mothers for vita butter bread was 247 g, and for vita tea bread was 196 g. The trans β‐carotene content of vita butter bread and vita tea bread were found to be 1.333 mg/100 g and 0.985 mg/100 g, respectively. The estimated trans‐β‐carotene intake was 3,293 μg/day (vita butter) and 1,931 μg/day (vita tea) based on the weighed bread intake, respectively, meeting 21% and 12% of the daily requirement (1,300 μg RAE/day) for lactating mothers, the life stage group with the highest vitamin A requirement. OFSP therefore could composite wheat flour to bake butter and tea bread, and will contribute to significant amount of dietary intake of vitamin A.

## 1 INTRODUCTION

Vitamin A deficiency (VAD) prevalence among mothers and children has internationally prompted the search for actions to combat it (Low, Walker, & Hijmans, 2001). These actions include supplementation, food fortification, biofortification, and diet diversification coupled with nutrition education (Berti, Faber, & Smuts, 2014). Combating this problem among those at risk will help decrease mortality and morbidity (Micronutrient Initiative, 2009), and measures to control the deficiency have been initiated in several countries including Ghana (WHO/FAO, 2006). VAD is severe in Ghana. About three fourth of preschool age children and one fifth of pregnant women are vitamin A deficient in Ghana (WHO, 2009). Low dietary intake of vitamin A or β‐carotene‐rich foods could be a major contributing factor to VAD prevalence in the country.

Bread consumption, however, is increasing in Ghana, and has been reported to be a staple food that takes the maximum cash expenditure within the food subgroups (Bonsi, Chibuzo, & Zibawa, 2014; Ellis et al., 1997). The most common types of bread consumed in Ghana include butter (prepared with a higher quantity of margarine), tea (prepared with less sugar and margarine), and sugar (prepared with a higher quantity of white sugar) (Tiimub, 2013). Almost all these bread types baked in Ghana are with 100% wheat flour that is processed from imported wheat due to unfavorable climatic conditions in the country for its cultivation (Nyumuah et al., 2012). In a previous study conducted by Bonsi et al. (2016), wheat‐only bread recipe, commonly referred to as sugar bread, was found to contain 3% of trans β‐carotene which is highly insignificant as compared to vita bread which was 17%. The contribution of the trans β‐carotene to the wheat‐only bread recipe may be due to the compulsory fortification of wheat flour, margarine, and milk, which are the basic ingredients for bread production in Ghana.

Orange‐fleshed sweet potato (OFSP) is one of the sweet potato varieties being promoted in sub‐Saharan Africa as a food‐based measure to complement other efforts in reducing the occurrence of VAD in this region (Kapinga, 2007; Low, Arimond, Labarta, Andrade, & Namanda, 2009; Tumwegamire, Kapinga, Zhang, Crissman, & Agili, 2004). Studies have indicated that consumption of boiled roots improved the vitamin A markers in adults and children (Hotz et al., 2012; Low et al., 2007; Tumwegamire et al., 2004). In spite of its nutritional value, OFSP is underutilized in Ghana compared with other root and tuber crops consumed (Baafi et al., 2015). OFSP has been found to be a good composite to wheat flour if pureed for bread production at 30% substitution based on Ghanaian consumer rankings for acceptability (Bonsi et al., 2014). Although the composite OFSP–wheat flour bread was found to be highly preferred, the concentration of vitamin A was not reported. Additionally, the 30% level substitution could be increased to make the composite OFSP–wheat flour bread contribute significant amount of dietary vitamin A. Therefore, compositing OFSP and wheat flour for bread with increased OFSP puree content is warranted. Incorporating OFSP puree in bread production would also provide a unique opportunity to create income for rural farmers, reduce the cost of wheat importation (Bonsi et al., 2014, 2016), and significantly contribute to VAD reduction (Hamed, Refai, Hussein, & El‐Samahy, 1973; Low & van Jaarsveld, 2008).

As suggested by other researchers (Hamed et al., 1973; Low & van Jaarsveld, 2008), refining bread recipes by the incorporation of sweet potato is not receiving the needed attention, although it has the potential to reduce the cost burden of wheat importation for wheat flour. Thus, OFSP composite bread could present double benefits: increasing income for farmers and bakers and improving dietary intake of vitamin A.

The objectives were to utilize OFSP puree in the development of two composite bread recipes and to assess their contribution to dietary intake of vitamin A using the dietary reference intake of lactating mothers.

## 2 MATERIALS AND METHODS

### 2.1 Source of raw materials and preparation of vita bread recipes

OFSP roots were acquired from a farm at Bontanga Irrigation Fields, in Northern Region of Ghana. Other ingredients such as wheat flour, margarine, salt, milk, yeast, nutmeg, and baking powder for baking were purchased from Tamale Central Market in Ghana. Baking was carried out at a commercial bakery Pakrozy Bakery in Tamale Metropolis, Northern Region, Ghana.

**Figure 1:**
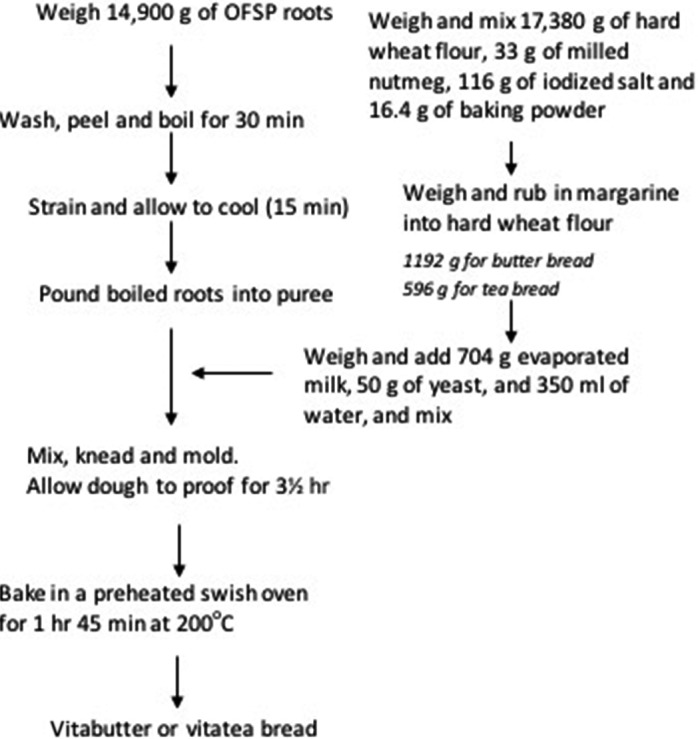
Flowchart for the preparation of vita bread

The OFSP roots were sorted to remove weevil infested ones, washed with tap water, peeled, and boiled for 30 min. The roots were then pounded into puree after cooling at room temperature. The wheat flour mixture comprising of hard wheat flour, nutmeg, salt, margarine, baking powder, and yeast was then added and mixed with the puree to form dough before baking (Figure 1).

### 2.2 Paired preference assessment of vita bread against conventional bread types

Sensory evaluation was conducted in Nyankpala, Tolon District and Sheshegu in Sagnarigu District of Northern Region, Ghana. The vita butter bread and vita tea bread were sliced and coded as 537 and 202, respectively. While conventional butter bread (628) and tea bread (334) samples were purchased from a retail shop in Tamale Metropolis.

A paired preference test was conducted to assess consumer preference for vita butter bread and vita tea bread against their respective conventional types. The coded samples were presented to 310 panelists for assessment. Ballot sheets were given to panelists to tick the bread sample they preferred based on the following attributes: appearance, aroma, sweetness, and overall degree of liking. Water was used as neutralizer between sample tasting.

### 2.3 β*‐*carotene assay

Vita bread samples were freeze‐dried, three replicates per vita butter and vita tea recipes, packaged in an opaque pulverized material before couriered to the Food and Nutrition Evaluation Laboratory, Nairobi, Kenya for the β‐carotene analysis. The samples were stored in a freezer at −20°C prior to analysis. Vita bread samples were removed from the freezer and allowed to thaw to room temperature in a room illuminated with yellow fluorescent lights. On average, 0.5 g of each sample was weighed (OHAUS, Biospec East Africa Ltd.) in duplicate in 25 ml screw‐capped glass tubes. This was followed by adding 6 ml of ethanol (Sigma‐Aldrich, ≥99.8% GC grade, USA) with 0.1% BHT (butylated hydroxytoluene). Each tube was vortexed (Scientific Industries, 0166, USA) for 1 min, and incubated in a water bath (SW23GB, JULABO) set at 85°C for 10 min. A 120 μl of 80% (w/v) potassium hydroxide (Sigma‐Aldrich, USA) was added to the tubes, vortexed for 1 min, and further incubated at 85°C for 5 min. The tubes were placed in ice to cool before addition of 3 ml deionized water. The tubes were then vortexed for 1 min, and 3 ml of hexane (Sigma‐Aldrich, ≥99.8% HPLC grade, USA) was added to each tube and further vortexed for 1 min. The samples were centrifuged (Eppendorf, Centrifuge 5810) at 800 x g for 10 min. The upper phase of hexane was pipetted using a Pasteur pipette into a separate 15 ml labeled test tubes. Extraction from the pellet was repeated thrice using 3 ml hexane, and the extract was combined in the 15 ml labeled test tubes. The combined hexane fraction was washed with 4 ml deionized water. The tubes were vortexed and centrifuged at 3,000 rpm for 3 min. The upper hexane phase was separated using Pasteur pipettes to new labeled test tubes. All the solvent was evaporated under nitrogen using N‐Evap machine (Organomation, Model OA‐8125) to complete dryness with a water bath set at 40°C. Dried extracts in tubes were reconstituted in 25 ml of methanol:tetrahydrofuran (85:15 v/v). Reconstitution was done by adding 10 ml of methanol:tetrahydrofuran ((85:15 v/v) to the tubes, mixing homogeneously for 1 min by a vortex, transferring to 25 ml volumetric flasks, and topping to the mark with 85:15 v/v methanol:tetrahydrofuran. The samples in flasks were vortexed for 1 min and sonicated for 30 s before transferring 1 ml to HPLC vials. A volume of 50 μl was injected into the HPLC. The HPLC system used was Waters 2695 separation module with 2996 PDA detector and a C30 carotenoid column (3 μm, 150 × 4.6 mm, YMC Wilmington, NC) utilizing a reverse phase gradient HPLC method. Mobile phase A constituted of methanol/tertbutyl methyl ether/water (85:12:3, v/v/v, with 1.5% ammonium acetate in the water) and mobile phase B constituted of methanol/tertbutyl methyl ether/water (8:90:2, v/v/v, with 1% ammonium acetate in the water). The total flow rate was 1 ml/min with each run going for 40 min. A quality control sample was run in every batch for quality control purposes. The amount for each trans β‐carotene component in the sample was calculated based on the area under the curve for each chromatogram.

### 2.4 Vita butter bread and vita tea bread intake assessment to calculate dietary vitamin A intake

Lactating mothers (*n *=* *50) were sampled purposively for this study. The study was carried out at Dungu in the Tamale Metropolitan District, Northern Region. A serving size of vita butter bread ranging from 236 to 520 g and vita tea bread of weight ranging from 178 to 527 g were given to these lactating mothers to consume. Leftovers were weighed to get the actual bread intake.

From the actual intake data of both vita butter and vita tea, the vitamin A profile of the vita bread were calculated using the Recommended Dietary Allowance (RDA) for vitamin A, which is 1,300 μg RAE/day for lactating mothers, the vulnerable group with the highest daily vitamin A requirement (Food and Nutrition Board, Institute of Medicine, & National Academies, 2004).

### 2.5 Statistical analysis

A two‐tailed binomial test was used to compare the preference scores for the developed vita bread types against their conventional types. Level of significance was estimated using binomial distribution for estimating significance in paired preference at 0.05 significance level as reported elsewhere by Roessler, Pangborn, Sidel, and Stone (1978). Based on this estimation, a minimum threshold of 170 as calculated out of the total number of respondents (*n *=* *310) was used to make the preferred counts statistically significant from the other.

Data generated from the food intake by the lactating mothers and trans β‐carotene analysis of the vita butter and vita tea bread recipes were compared using the two‐sample *t*‐test procedure in Minitab version 16.2^TM^ (Minitab Inc., State college, PA, USA) at 0.05 significance level.

## 3 RESULTS

### 3.1 Development of vita bread

**Figure 2:**
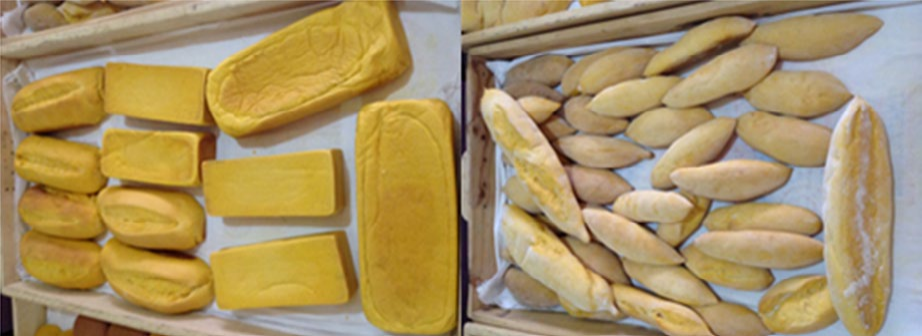
Developed vita bread types

OFSP puree was used to substitute 46% wheat flour for both vita butter and vita tea bread production using previous recipes of composite OFSP–wheat flour bread (Bonsi et al., 2016). The difference between vita butter bread and vita tea bread developed were the quantity of margarine used and their shapes after molding per Ghanaian style for tea bread and butter bread production (Figure 2). Also, the quantity of yeast was increased to speed up the proofing time. No refined sugar was added. The incorporation of 46% puree for the developed vita butter and vita tea recipes was 53% and 44% increase over the earlier recipes published by Bonsi et al. (2014, 2016), respectively. The OFSP‐based bread types had favorable aroma and firmness as desired on the Ghanaian market (Peter Koomson, personal communication, Pakrozy Bakery, 10 February 2016).

### 3.2 Consumer ranking for vita bread versus conventional types

**Figure 3:**
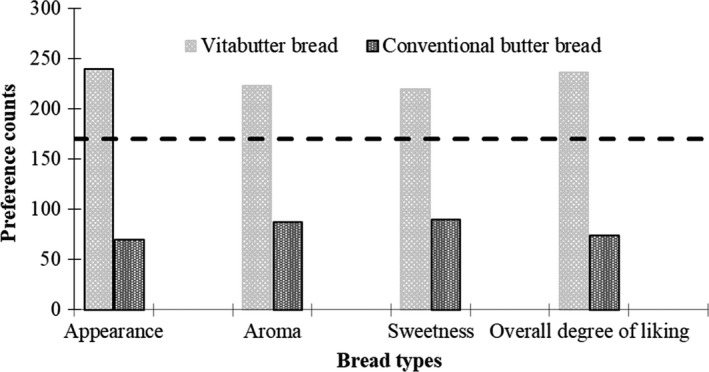
Preference for vita butter bread as compared to conventional butter bread

**Figure 4:**
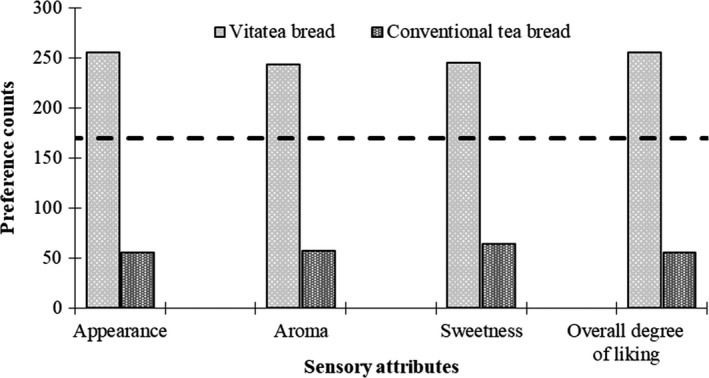
Preference for vita tea bread as compared to conventional tea bread

Results on the preference of vita bread types against their respective conventional bread types are presented in Figures 3 and 4. From the results of the paired preference test conducted, vita butter and vita tea bread were found to be most preferred over existing ones in terms of appearance, aroma, sweetness, and overall degree of liking. The preference for vita butter bread was about two to three times more than that of the conventional butter bread. Similarly, the preference for vita tea bread was three to four times more than that of the conventional tea bread.

### 3.3 β*‐*carotene assay

**Figure 5:**
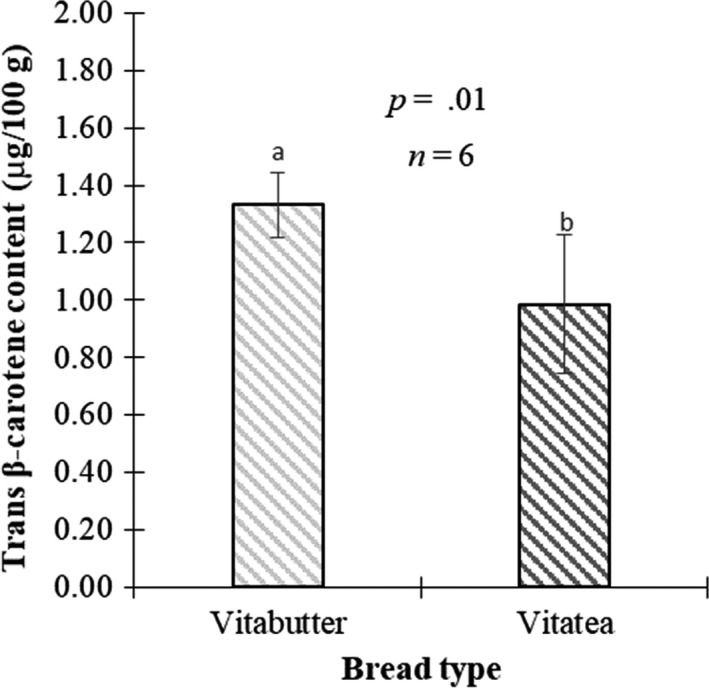
Trans β‐carotene content of vita bread recipes

The trans β‐carotene content of vita bread is shown in Figure 5. Using the trans β‐carotene intake of wheat white bread, which is 260 μg/100 g (Bonsi et al., 2016), vita butter bread and vita tea bread exceeded the amount in this wheat white bread by 412% and 279%, respectively. However, the vita butter bread had significantly higher trans β‐carotene (1.3 times, *p *=* *.01) than that in vita tea bread.

### 3.4 Dietary vitamin A contribution from the consumption of vita bread types by lactating women

**Figure 6:**
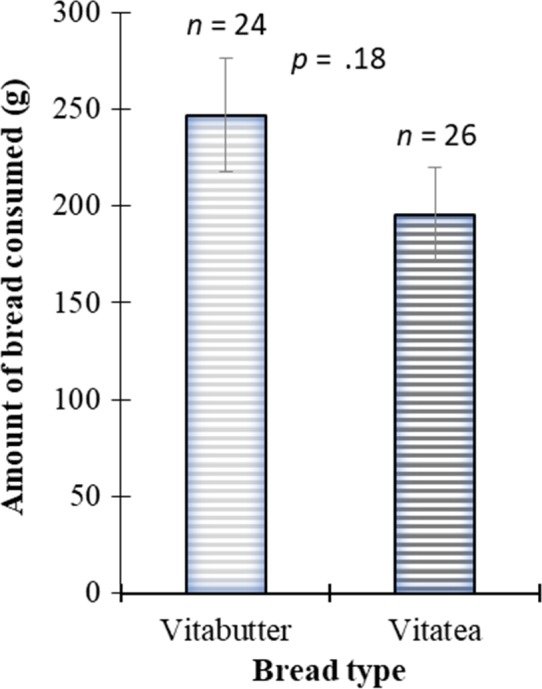
Mean of the dietary intake of OFSP‐based bread by lactating women

The mean intake of bread by lactating mothers were 247.10 ± 144.87 (g) for vita butter bread and 195.60 ± 122.76 (g) for vita tea bread (Figure 6). However, the quantities consumed by the lactating mothers were not statistically different (*p *=* *.8).

## 4 DISCUSSION

In this study, it has been shown that OFSP puree can be incorporated into wheat flour at higher level than the 30% (Bonsi et al., 2014) or 32% (Bonsi et al., 2016) previously reported. The exclusion of sugar and the higher substitution of OFSP puree in these recipes suggest more profit for bakers as the proportion of wheat flour has been reduced. This will help reduce the importation of wheat for flour production. However, economic analysis study is required to substantiate the proposed higher profit margin for the vita bread recipes.

The higher consumer preference for vita butter bread and vita tea bread than conventional 100% wheat flour bread types may be due to the inherent color, flavor, and sweetness of OFSP (Leighton, 2007). The consumer preference data from this present study is similar to previous work conducted in Ghana (Bonsi et al., 2014, 2016) and elsewhere in Africa (Low & van Jaarsveld, 2008) on bakery products based on sweet potato. This suggests a high patronage of vita butter bread and vita tea bread if they are introduced into the Ghanaian markets.

Based on the trans β‐carotene level and its conversion ratio to the daily RDA of vitamin A and the bread intake data, the vita butter bread and vita tea bread will, respectively, meet 21% and 12% of 1,300 μg/day of the daily RDA of vitamin A of lactating mothers especially between 15 and 25 years. Furthermore, the vita bread intake data and the amount of trans β‐carotene in these composite bread types suggest that they could be crucial in addressing VAD in Ghana, contributing to at least 12% of the daily requirements of vitamin A needs by lactating mothers, which indicate that it is a significant source of dietary vitamin A. This suggestion is based on earlier findings that bread has become a staple food in Ghana (Komlaga, Glover‐Amengor, Dziedzoave, & Hagan, 2012). Therefore, initiatives aimed at improving the vitamin A status of vulnerable groups in Ghana using food‐based measures should adopt these vita bread recipes. This will help reduce the cost of other measures such as supplementation and food fortification. In addition, vita bread would be of great importance to children because it will meet their dietary vitamin A requirement which is about a quarter of that of lactating mothers.

The increase in trans β‐carotene in the vita bread types as compared to wheat white bread is due to the OFSP puree used. Also, higher amount of trans β‐carotene in the vita butter bread as compared to that of the vita tea bread might be as a result of the increase in the margarine content in the recipe, as vitamin A is used to fortify margarine in Ghana (IMACE, 2004). It is however possible that the trans β‐carotene content of conventional butter bread will be higher than other types of conventional bread, although not significant as compared to vita bread recipes.

The bottleneck associated with the consumption of OFSP by Ghanaians because it is sweet (Baafi et al., 2015) would be addressed by compositing OFSP and wheat flour to produce bread. This will increase utilization of OFSP roots.

Because of the high demand of bread in Ghana, the vita bread recipes are likely to create a market pull for OFSP roots, and consequently ensuring income for rural farmers as well as bakers and sellers who will invest in this important crop. This is because there would be a high demand for OFSP puree by bakers to produce these bread types.

The success of vita bread in addressing VAD would, however, depend on the availability of roots all year round, and this can be achieved through stagger planting and storage. Unless there is access to irrigation facility and appropriate storage structure, availability of roots all year round will be challenging.

## 5 CONCLUSION

OFSP, a high β‐carotene crop, was used to develop puree for bread production. Recipes developed could be a viable alternative to 100% wheat flour recipes for health and wealth. The vita bread types therefore may contribute significant amounts of dietary vitamin A in the Ghanaian diet especially that of lactating mothers. Further studies of glycemic index and effect on serum retinol or vitamin A biomarkers are warranted.

## CONFLICT Of INTEREST

The authors declare no conflict of interest.
